# Pectointercostal fascial plane block for rescue pain management of traumatic sternal fracture following inadequate thoracic epidural block: a case report

**DOI:** 10.1093/jscr/rjac073

**Published:** 2022-03-30

**Authors:** Michael Hsu, Sudhakar Kinthala, Jordan Huang, Neel Kapoor, Poovendran Saththasivam, Burdett Porter

**Affiliations:** Department of Anesthesiology, Guthrie Clinic, Sayre, PA, USA; Department of Anesthesiology, Guthrie Clinic, Sayre, PA, USA; Department of Anesthesiology, Guthrie Clinic, Sayre, PA, USA; Department of Anesthesiology, Guthrie Clinic, Sayre, PA, USA; Department of Anesthesiology, Guthrie Clinic, Sayre, PA, USA; Department of Anesthesiology, Guthrie Clinic, Sayre, PA, USA

## Abstract

Adequate pain control after multisystem trauma including the chest wall is essential for improved patient outcomes, especially with sternum and rib fractures. The thoracic epidural is considered the gold standard in pain management of thoracic injury; however, failure or patchy epidural is not uncommon. Pectointercostal fascial plane block (PIFB) is regularly used in cardiac surgery to provide analgesia to the anterior chest wall; however, there are few reports of PIFB being used as a primary block for the management of thoracic injuries. We present a case in which PIFB was used as a rescue block for the successful management of sternal pain following patchy thoracic epidural block in a patient with thoracic polytrauma.

## INTRODUCTION

Adequate analgesia is paramount for the management of traumatic thoracic injury and mitigates the negative effects of chest wall pain on pulmonary function parameters [1]. Opioids are commonly used for pain control in trauma settings; however, complications such as oversedation, respiratory depression, delirium, bowel dysfunction and abuse potential are well known [2]. 2016 Eastern Association for the Surgery of Trauma guidelines recommend thoracic epidural analgesia (TEA) as the preferred modality of analgesia for nonoperative management of bilateral rib fractures [3]. However, failure of epidural analgesia can occur in up to 30% of cases [4]. In such situations, additional regional anesthetic is a potential option to achieve optimal pain control. The pectointercostal fascial plane block (PIFB) provides analgesia to the anterior chest wall via innervation by the anterior cutaneous branches of the intercostal nerves and avoids major bleeding risk with heparinization [5]. Its application in the trauma setting has not been widely studied. The literature is limited to a few case reports where PIFB is used as the primary block for managing sternal fractures [6]. We present the first case in which PIFB was used as a rescue block for successful management of sternal pain after patchy thoracic epidural.

Patient permission was obtained for publication of this case report.

## CASE REPORT

**Figure 1:**
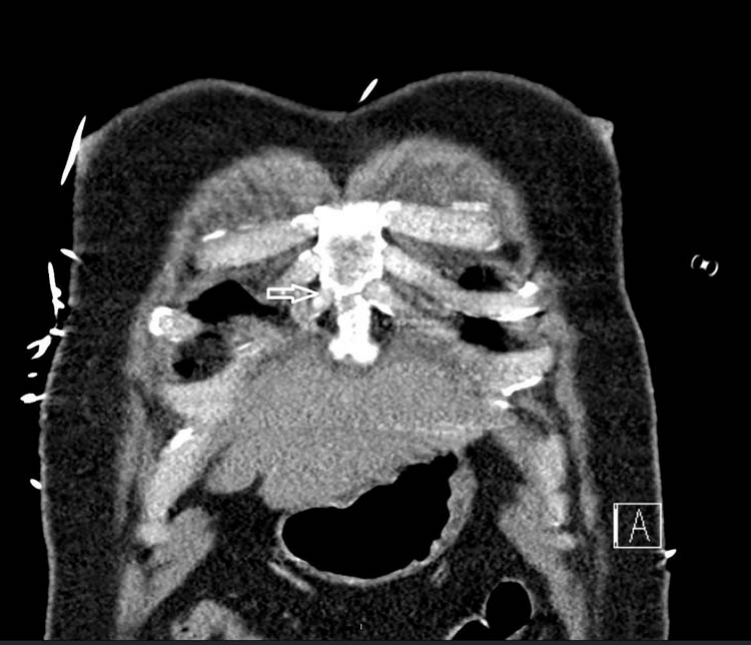
Computed tomography scan showing nondisplaced sternal body fracture (arrow).

A 50-year-old 105 kg Caucasian man (BMI 32) without prior medical history presented to the trauma bay after motor vehicle accident. Physical examination in the trauma bay and computed tomography revealed fractures of right anterior ribs 2, 3, 4, 5, 6, 7 and 8, right posterior ribs 5, 6 and 8, lateral rib 9 and left anterior ribs 7 and 8, along with pulmonary contusions, left-sided hemothorax, sternal body fracture (Fig. 1) and a grade 2 liver laceration. We decided to manage the patient’s injuries nonoperatively. Under strict aseptic conditions, a thoracic epidural was placed at T5. Despite adequate pain relief from his bilateral rib fractures, significant sternal pain from the fracture persisted. Due to difficult positioning and challenges with patient cooperation, we decided to place a PIFB block as a rescue for the inadequate thoracic epidural.

Under strict aseptic conditions, we placed bilateral PIFB under ultrasound guidance using a linear ultrasound probe oriented craniocaudally 2 cm lateral to the edge of the sternum on the right side over ribs 4 and 5, and the same procedure was repeated on the left side. Immediate relief of sternal pain was obtained after placement with bilateral boluses of 10 ml 0.5% ropivacaine. Catheters were placed to continuously deliver 5 cc of 2 μg fentanyl and 0.1% ropivacaine every hour. The patient reported a numerical rating pain score (NRS) of 8 at rest and 10 with coughing and movement before bilateral PIFB placement but an NRS of 0–2 at rest and 4–5 with activity afterwards. Improvement in respiratory function was evident from 300 cc to 1000 cc on incentive spirometry. The PIFB catheters were removed on postoperative day (POD) 4, and the thoracic epidural catheter was removed on POD 5. No complications were noted during the hospital stay and on follow-up.

## DISCUSSION

Regional pain blocks such as PIFBs offer an alternative method to optimize postoperative recovery in patients with sternotomy pain and in the trauma setting. PIFB was introduced by de la Torre as a tool to control mastectomy-associated pain [5]. Pectoral nerve blocks can also provide pain relief at the medial anterior chest wall [7]; however, PIFB may offer more precise analgesia due to local anesthetic being injected between the pectoralis major and internal intercostal muscles [5]. The transversus thoracic plane (TTP) block is considered a deeper version of the PIFB and can provide a similar degree of analgesia; however, placement of the needle tip in the fascial plane between the internal intercostal and transverse thoracic muscles is more technically challenging [8]. PIFB can also be considered a safer option based on the anatomical sites of injection, avoiding major vasculature and viscera. Rare complications include pneumothorax, vessel injury, hematoma and infection [8].

The efficacy of PIFB in modulating chest wall pain has previously been described post-sternotomy after coronary artery bypass grafting (CABG). Liu *et al*. reported a case of an elderly CABG patient who experienced immediate relief following PIFB, requiring reduced opioid consumption [9]. Moreover, Kumar *et al*. were able to demonstrate in a randomized controlled trial that in cardiac patients, PIFB provides equal pain relief as an erector spinae plane block with normal tidal volume breathing [10].

Literature regarding the use of PIFB block in trauma patients is limited. Burns *et al*. described successful pain management in three trauma patients, all with anterior rib and sternal fractures [6]. However, Burns *et al*. opted to perform a PIFB as the initial option, whereas we initially chose a thoracic epidural for pain management to account for multiple bilateral fractures. We introduced PIFB as a rescue for a patchy epidural as the patient’s sternal pain was still uncontrolled despite adequate pain relief from rib fractures. Due to our prior difficulty with patient positioning, we opted to maintain the original epidural with PIFB supplementation. We opted for PIFB over TTP due to greater ease of placement and less risk of potential complications.

This case report has a few limitations. We are unsure of the cause of adequate pain control to the entire chest wall except the sternum. Second, the patient’s polytrauma pain may be multifactorial with multiple sources, and the perception of pain relief from our TEA and PIFB may thus be affected.

## CONCLUSION

PIFB is effective for managing pain due to sternal and anterior thoracic injuries. It can be used as a primary option or as a rescue option with a patchy epidural for managing anterior chest wall and sternal pain.

## CONFLICT OF INTEREST STATEMENT

None declared.

## FUNDING

None.
